# Comprehensive Evaluation and Analysis of the Mechanism of Cold Tolerance Based on the Transcriptome of Weedy Rice Seedlings

**DOI:** 10.1186/s12284-019-0363-1

**Published:** 2020-02-13

**Authors:** Bing Han, Xiaoding Ma, Di Cui, Yanjie Wang, Leiyue Geng, Guilan Cao, Hui Zhang, Longzhi Han

**Affiliations:** 1 Institute of Crop Sciences, Chinese Academy of Agricultural Sciences, Beijing, 100081 China; 2 Coastal Agriculture Institute, Hebei Academy of Agricultural and Forestry Sciences, Tangshan, 063299 China

**Keywords:** Weedy rice, Morphological index, Physiological indicators, Transcriptome, Differential expression

## Abstract

**Abstract:**

In this study, the cold-tolerance capacity of 133 varieties of weedy rice was evaluated based on the comprehensive evaluation index D, with Kongyu 131 used as a cold-tolerant control. A total of 39.8% of the 133 varieties were considered ‘strong’, indicating that weedy rice populations indeed have relatively strong cold-tolerance capacity as a whole, and the robust cold-tolerant varieties WR29 and WR157 were identified. Regression analysis showed that the metrics including the nitrogen recovery index, superoxide dismutase (SOD) content and malondialdehyde (MDA) content correlated significantly (*P* < 0.05) with cold tolerance and could be used as indicators of cold tolerance. On the basis of a transcriptome analysis of WR157, a robust cold-tolerant variety identified in this study, a total of 4645 putative DEGs were identified in treated groups compared to the control groups, with 2123 upregulated DEGs and 2522 downregulated DEGs. All upregulated DEGs were enriched on 1388 terms, all downregulated DEGs were enriched on 1566 terms; 911 of the 2123 upregulated DEGs fell into 98 KEGG categories and 1103 of the 2522 downregulated DEGs were in 115 categories. Further analysis showed that GO:0019740 and GO:0006808 are involved in nitrogen utilization; GO:0009269 and GO:0009414 are related to the stress response; and GO:0016491 and GO:0016614 are related to oxidoreductase activity.

**Background:**

Weedy rice (*Oryza*) is a related pest species of cultivated rice (*Oryza sativa* L.) that has strong abiotic stress resistance; however, the comprehensive mechanism governing its cold tolerance is poorly understood.

**Conclusion:**

Our comprehensive evaluation based on five morphological indices and nine physiological indicators revealed outstanding levels of cold-tolerance capacity among weedy rice varieties from different regions and revealed some terms related to cold tolerance via transcriptome analysis. Our results underscored the reliable evaluation methods for additional cold tolerance studies and revealed several genes related to cold tolerance, which will help researchers breed cultivated rice varieties to increase their cold-tolerance capacity. These traits have the ability to increase seedling survival rate and growth, as well as future yields.

## Background

Rice (*Oryza sativa* L*.*) is one of the most important staple crops. Weedy rice is a type of rice that produces fewer grains and occurs as a pest species within fields of cultivated rice. Weedy rice has strong resistance to stress (cold, drought and salt), while cold damage severely influences the growth and yield of cultivated rice. Cold stress has demonstrated negative impacts on rice yields in 25 countries worldwide (Cruz et al. 2013), and the average yield loss annually is 3–5 billion kg, threatening food security and economic development. In Northeast China, chilling damage to rice has increased more than 10-fold since the 1950s (Chen et al. 2015). Despite these impacts, little is currently known about the mechanism of cold tolerance, which plays an important role in preventing and reducing cold damage.

Studies of cold tolerance use evaluation indices to identify the cold-tolerance capacity of rice crops (Kuroki et al. 2007, Ma et al. 2015, Li et al. 2018). Yang et al. (2010) used the cold response index (CRI) and seed setting rate (SSR) to evaluate cold tolerance. Xiao et al. (2014) identified cold-tolerance capacities at the bud stage via the survival rate (SR). Lakra et al. (2015) studied the chlorophyll contents under abiotic stress conditions. Changes in the nitrogen content of leaves represent an evaluation standard for adversity (Cao et al. 2018), while leaf withering degree (LWD) is an index for evaluating cold tolerance and drought resistance in stress studies (Han et al. 2018).

Some physical or biological signatures can also be used in assays of cold tolerance. The activity of antioxidant enzymes and malondialdehyde (MDA) content can be used to detect cold stress or tolerance in crop plants. For example, superoxide dismutase (SOD) and catalase (CAT) can protect cells from damage, the malondialdehyde (MDA) content reflects the degree of damage under stress conditions, and the abscisic acid (ABA) content is related to Ca^2+^ present under physical or chemical stress. Increased proline (Pro) can enhance cold tolerance responsiveness, while chlorophyll content can dramatically decline under adverse conditions (Li et al. 2015). Under stressful conditions, triosephosphate isomerase (TPI) is regulated by cysteine, and ATP is consumed excessively (Ma et al. 2016).

Plants are sessile organisms and have evolved a series of mechanisms to adapt to cold environments throughout their long-term evolution. Cold tolerance and cold acclimatization are the two different mechanisms used by plants under cold-stress conditions. Cold tolerance is considered the intrinsic ability to survive when plants confront cold stress; cold tolerance is the result of complex metabolic regulatory activity and is a morphologically adaptive process, during which the expression of proteins related to signal transduction, gene expression and damage repair are activated. Cold acclimatization is defined as the accessorial potential via changes in substances such as physicochemical components, osmolytes (soluble sugars and alcohols) and various nitrogenous compounds (proline and glycine betaine) (Somerville 1995).

The typical symptoms of low-temperature-stress conditions include accumulations of metabolites, changes in carbohydrate metabolism, increases in reactive oxygen species (ROS), decreases in photosynthesis, and reductions in stomatal conductivity. There are two signal transduction pathways that respond to low-temperature stress. One is the ABA-dependent pathway, in which ABA promotes the expression of cold tolerance genes under adverse conditions. In ABA-independent response pathways, CBF/DREB1 (C-repeat-binding factor/dehydration-responsive element binding factor) is an important transcription factor that responds to cold-temperature signals (Bartels and Sunkar 2005, Farooq et al. 2009). Cold-regulated proteins (CORs), dehydrins, and TFs (AREBs or ABFs) in the ABA-dependent pathway are induced by the production of osmolytes and ROS (Thomashow 1999, Choi et al. 2000, Uno et al. 2000, Agarwal et al. 2006, Peng et al. 2008, Moraes de Freitas et al. 2019). These stress-induced proteins ultimately quench reactive oxygen species to stabilize membrane phospholipids and maintain ionic homeostasis. In recent years, due to advances in cell and molecular biology, the study of cold tolerance has achieved rapid development. In particular, transcriptome analysis has proven to be a critical method for studying stress tolerance in crops. For example, cold tolerance at the rice germination stage has been described by RNA-seq methods in Indica genotypes (Dametto et al. 2015). Comparative transcriptomics has been used to measure cold stress responsiveness in two rice varieties (Zhang et al. 2012) and to detect cold, iron, and salt stress in rice plants (do Amaral et al. 2016). Transcriptome analysis has been used to analyze salt stress responsiveness at the seedling stage in Dongxiang wild rice (Zhou et al. 2016) and to detect phosphorus stress responsiveness in seedlings of Dongxiang wild rice (Deng et al. 2017, Deng et al. 2018a, 2018b). Guan et al. (2018) analyzed the tolerance of weedy and cultivated rice with transcriptomic profiling.

In our study, five morphological indices were used to evaluate the cold tolerance capacity of 133 rice varieties. Nine physiological indices were used to verify the changes in cold-tolerance capacity of six selected varieties from 133 tested varieties, and transcriptome analysis of one representative variety (WR157) was used to identify the cold tolerance and analyze the mechanism of cold tolerance. Our findings provide information for further research direction for examining the mechanism of cold resistance in cultivated rice.

## Results

### Comprehensive Evaluation of Cold Tolerance Based on 5 Morphological Indices

In our study, the SR (seedling survival rate), CRI (chlorophyll content recovery index), NRI (nitrogen content recovery index), and LWD (leaf withering degree) recovery indices were measured on the 7th day after recovery growth (LWD7 and RLWD7) to evaluate the cold damage resilience of different rice varieties.

Via correlation analyses between each index and cold-tolerance capacity, significantly correlated indicators were selected to evaluate the cold tolerance of each variety. Though these methods decreased the number of evaluation indices, due to the difference in cold-tolerance mechanisms in different varieties, information about each evaluation index can overlap; therefore, it is very difficult to correctly evaluate the cold tolerance of each variety using these indices. However, PCA (principal component analysis) can reduce the number of the variables (indices) to several potential factors and assure that the information of the variables (indices) is not lost or that little is lost. The information concerning the several factors highly summarizes and represents a large amount, and it not only reduces the number of the variables but also reestablishes the inner link among all the variables.

In this paper, PCA (principal component analysis) of 5 morphological indices generated two comprehensive indices, CI (1) and CI (2), which had contribution rates of 54.545% and 32.01%, respectively. The cumulative contribution rate reached 86.555% (Table 1). The two comprehensive indices were calculated for each variety (Additional file 1: Table S1, Additional file 2: Table S2).

**Table 1:** Coefficients of comprehensive index CI(x) and contribution rate

Index	LWD7	RLWD7	SR	CRI	NRI	CR(%)
CI(1)	− 0.305	− 0.264	0.258	0.26	0.265	54.545
CI(2)	0.171	0.317	−0.35	0.436	0.427	32.01

*LWD7* leaves withered degree at 7th day of clod treatment, *RLWD7* LWD for recover growth fat 7th day, *SR* seedling rate, *CRI* chlorophyII recover index, *NRI* nitrogen recover index, *CR* contribution rate

As shown in Additional file 3: Table S3, the subordinate function μ values for the comprehensive scores of each sample were calculated according to the Eq. (1) (refer to the Methods section), and the subordinate function μ value can reflect the cold tolerance of each sample. For the same comprehensive scores, the cold tolerance could be evaluated according to the subordinate function μ value. The weight factor of each variety was then calculated according to Eq. (2) (refer to the Methods section), which suggested the degree of importance of the comprehensive index. According to the calculation results, the weight function *w*_*i*_ was calculated and represents the relative importance for the ith comprehensive index, and *p*_*i*_ represents the contribution for the ith comprehensive index. The comprehensive weights for the two comprehensive indices CI (1) and CI (2) were 0.630 and 0.369, respectively, the comprehensive weight was 1. Via the subordinate function μ and weight factor *w*_*i*_, according to Eq. (3), the comprehensive evaluation value or recovery index D (Additional file 4: Table S4) for cold tolerance at the seedling stage was calculated. Moreover, according to the D value, the cold-tolerance capacity of every variety can be ranked; the larger the value of D is, the stronger the cold tolerance of the samples.

Among the examined varieties, WR29 had the greatest *D* value (0.8328), the value for WR157 was 0.8219, the value for Kongyu 131, the cold-tolerant control, was 0.8181, and the value for the variety WR165 was 0.7994, suggesting that the weedy rice varieties WR29 and WR157 have a greater cold-tolerance capacity than the traditional cold-tolerant control Kongyu 131. Generally, when *D* > 0.8, the cold-tolerance capacity of a given variety is very strong, when 0.8 > *D* > 0.5, the cold-tolerance capacity is considered strong, 0.5 > *D* > 0.2 indicates a moderate cold tolerance, and *D* < 0.2 is considered weakly cold tolerance. The *D* value for 78.9% of the varieties was greater than 0.2, and the *D* value for 39.8% of the varieties was greater than 0.5 (Additional file 4: Table S4). These findings suggest that the weedy rice populations as whole have a relatively strong cold-tolerance capacity. Regression analysis showed that the NRI correlates significantly with the cold tolerance of weedy rice (*r* = 0.926, *P* < 0.01), which suggests that the leaf NRI is involved in the mechanism of cold resistance and that the NRI could be a critical index of cold tolerance evaluation.

### Comprehensive Evaluation of Cold Tolerance Based on Physiological Indicators

In this paper, nine physiological indices including superoxide dismutase (SOD) content, catalase (CAT) content, triosephosphate isomerase (TPI) content, malondialdehyde (MDA) content, proline (Pro) content, ATP synthase (ATPase) content, abscisic acid (ABA) content, chlorophyll (Chl) content, and glutathione (GSH) content were used to evaluate cold-tolerance capacity.

**Fig. 1:**
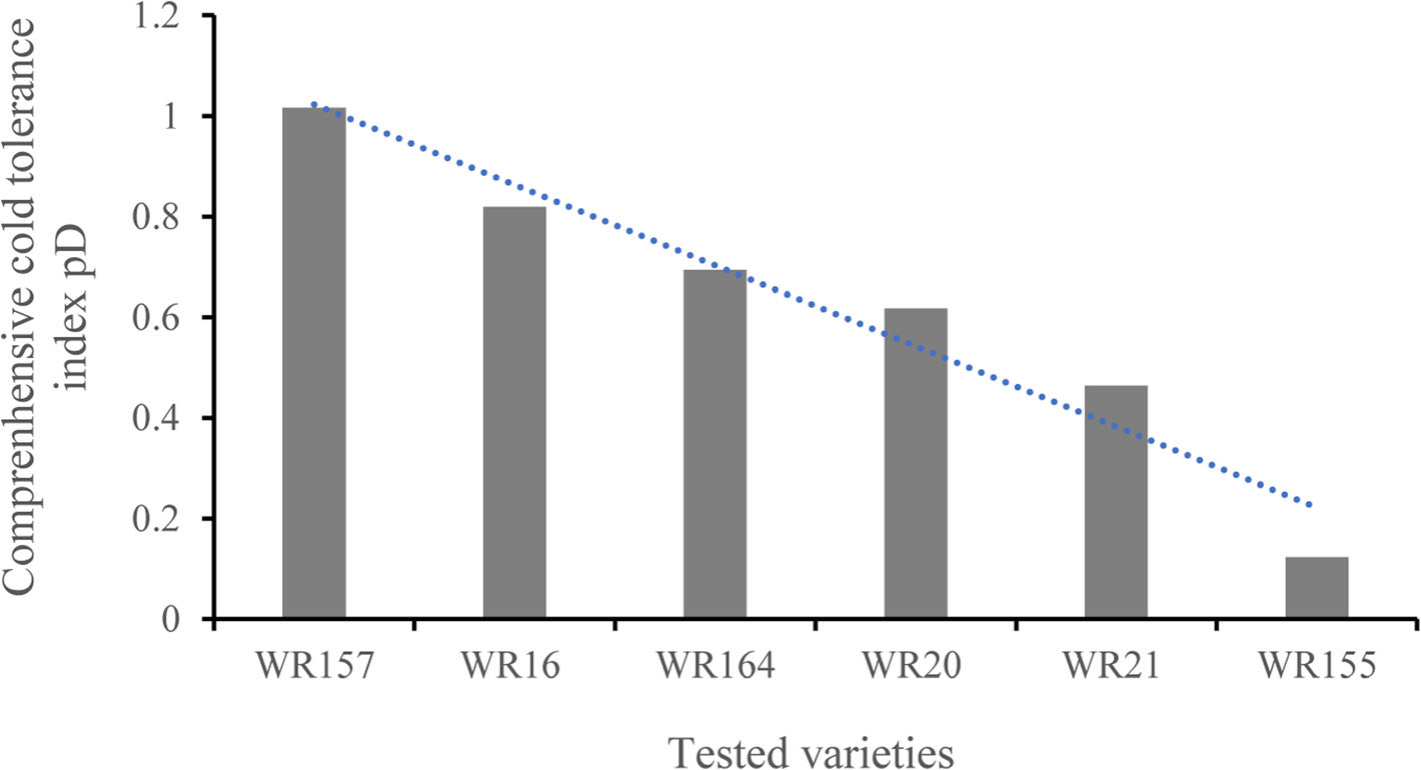
The changed trend of cold-tolerance capacity of 6 select varieties based on 9 physiological indicators. The *p*D value based on the 9 physiological indicators, and the meaning is equal to the significance of the D value based on the 5 morphological indices. 9 physiological indicators: SOD: superoxide dismutase content, CAT: catalase content, TPI: triosephosphate isomerase content, MDA: malondialdehyde content, Pro: proline content, ATPase: ATP synthase content, ABA: abscisic acid content, Chl: chlorophyll content, GSH: glutathione content

**Fig. 2:**
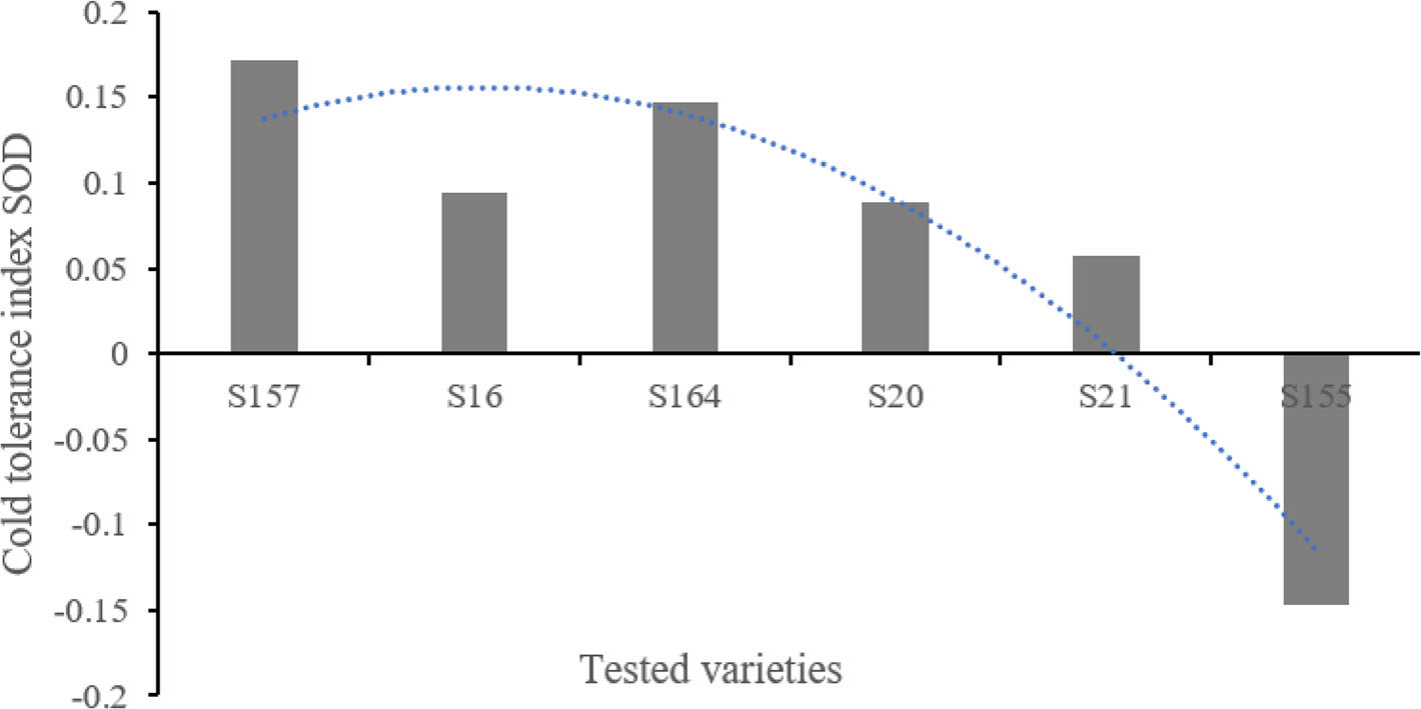
SOD changes in six selected weedy rice varieties. The changes in superoxide dismutase (SOD) content are shown along with the cold-tolerance capacity of the different varieties. The positive value represents a relative increase compared with the control group (untreated group), and the negative value represents a relative decline compared to the control group (untreated group), S157 (WR157), S16 (WR16), S164 (WR164), S20 (WR20), S21 (WR21) and S155 (WR155) represented 6 diffferent varieties

**Fig. 3:**
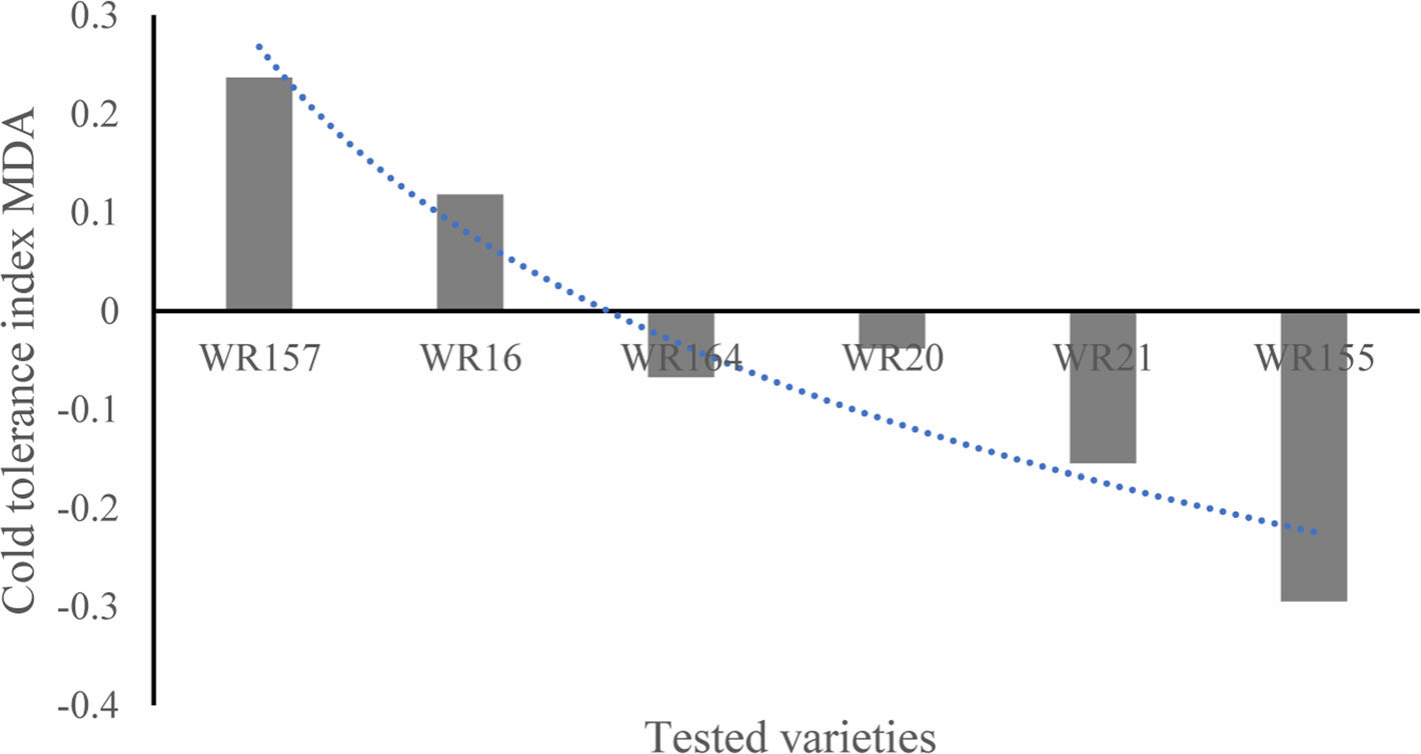
MDA changes in six select weedy rice varieties. The changes in malondialdehyde (MDA) value are shown along with the cold-tolerance capacity of different varieties. The positive value represents a relative increase compared with the control group (untreated group), and the negative value represents a relative decline compared to the control group (untreated group)

PCA of the 9 physiological indicators for 6 cold-tolerant varieties (Additional file 5: Table S5) revealed three comprehensive indices p CI (1), p CI (2) and p CI (3), which had contribution rates of 46.789%, 28.146%, and 18.904%, respectively. The cumulative contribution rate reached 93.84% (Table 2). According to Eq. (1), the subordinate function values *pμ*(1), *pμ*(2), and *pμ*(3) were calculated. According to Eq. (2), the weight function p W was calculated, and the comprehensive weights for the three comprehensive indices were 0.499, 0.300, and 0.201, respectively. According to Eq. (3), the comprehensive cold-tolerance index *pD* Was calculated (Table 3, Fig. 1). *pD* reflected the trend of changes in cold tolerance capacity of six tested varieties: the WR157 variety had the maximum relative cold tolerance index, while WR155 had the minimum relative cold tolerance index (Fig. 1). The regression equation *pD* = 0.585 + 1.1061*x*_4_ + 1.065*x*_1_ was constructed (*x*_4_ represents the MDA physiological indicator, and *x*_1_ represents the SOD physiological indicator). The results suggested that among the 9 considered physiological indicators, SOD activity and MDA content have important significance in the evaluation of cold tolerance (Figs. 2 and 3). As shown in Figs. 2 and 3 showed, SOD content and MDA content reflected the changes of six tested varieties compared to the control WR157CK. In the treated WR157 variety, the increased content of SOD and MDA was the highest, and in WR155, the content of SOD and MDA declined relative to other varieties. The varieties WR157 and WR16 have high cold tolerance, while WR164, WR20, and WR21 have moderate cold tolerance (Table 3). The SOD physiological indicator was used to identify the cold-tolerance capacity of the 6 tested varieties, and the results aligned with those obtained from the optimal equation. There was a significant positive correlation between *pD* the SOD value (*r* = 0.971, *P* < 0.01, Fig. 2) and between *pD* and the MDA value (*r* = 0.921, *P* < 0.01, Fig. 3).

**Table 2:** The coefficients of comprehensive index p CI (x) and contribution rate

Index	SOD	CAT	GSH	MDA	ATPase	Pro	Chl	ABA	TPI	p CR (%)
p CI (1)	0.201	−0.125	0.145	0.108	−0.097	0.23	−0.236	0.089	0.151	46.789
p CI (2)	0.197	−0.241	−0.157	0.348	0.3	−0.081	0.008	−0.212	− 0.106	28.146
p CI (3)	0.012	0.347	0.248	−0.019	0.26	0.061	−0.003	−0.44	0.374	18.904

**Table 3:** The different value of comprehensive evaluation of each variety

Varieties	p CI (1)	p CI (2)	p CI (3)	p μ (1)	p μ (2)	p μ (3)	p D	p VP
WR157	0.707	1.073	−1.059	0.922	0.928	0.000	1.016	1.020
WR16	−0.540	1.252	1.509	0.440	1.000	1.000	0.819	0.811
WR164	0.910	−0.433	0.106	1.000	0.325	0.454	0.694	0.670
WR20	−0.127	0.085	−0.873	0.600	0.532	0.072	0.618	0.639
WR21	0.732	−1.245	0.783	0.931	0.000	0.717	0.464	0.481
WR155	−1.682	−0.733	−0.466	0.000	0.205	0.231	0.123	0.116
p W				0.499	0.300	0.201

In this study, we provided a new way of categorizing cold tolerance using comprehensive indexes D and *p*D for the morphological and physiological traits, respectively. Through regression analysis, this approach allows for the identification of traits that correlate most significantly with cold tolerance in weedy rice, and to further correlate those traits with potential cold tolerance pathways based on transcriptome analysis.

### Cold Tolerance Analysis Based on Transcriptomics at the Seedling Stage

The strong cold-tolerant variety WR157 was selected, and at 3 d after cold stress, RNA was extracted from leaves of plants in the WR157CK (control group) and WR157T (treatment group) for transcriptome analysis; RNA was extracted from leaves of plants in the WR157CK (control group) and WR157T (treatment group) for three biological replicates and the extracted RNA from each biological replicate was mixed into one RNA sample in a mass ratio of 1:1:1 for the transcriptome analysis.

**Fig. 4:**
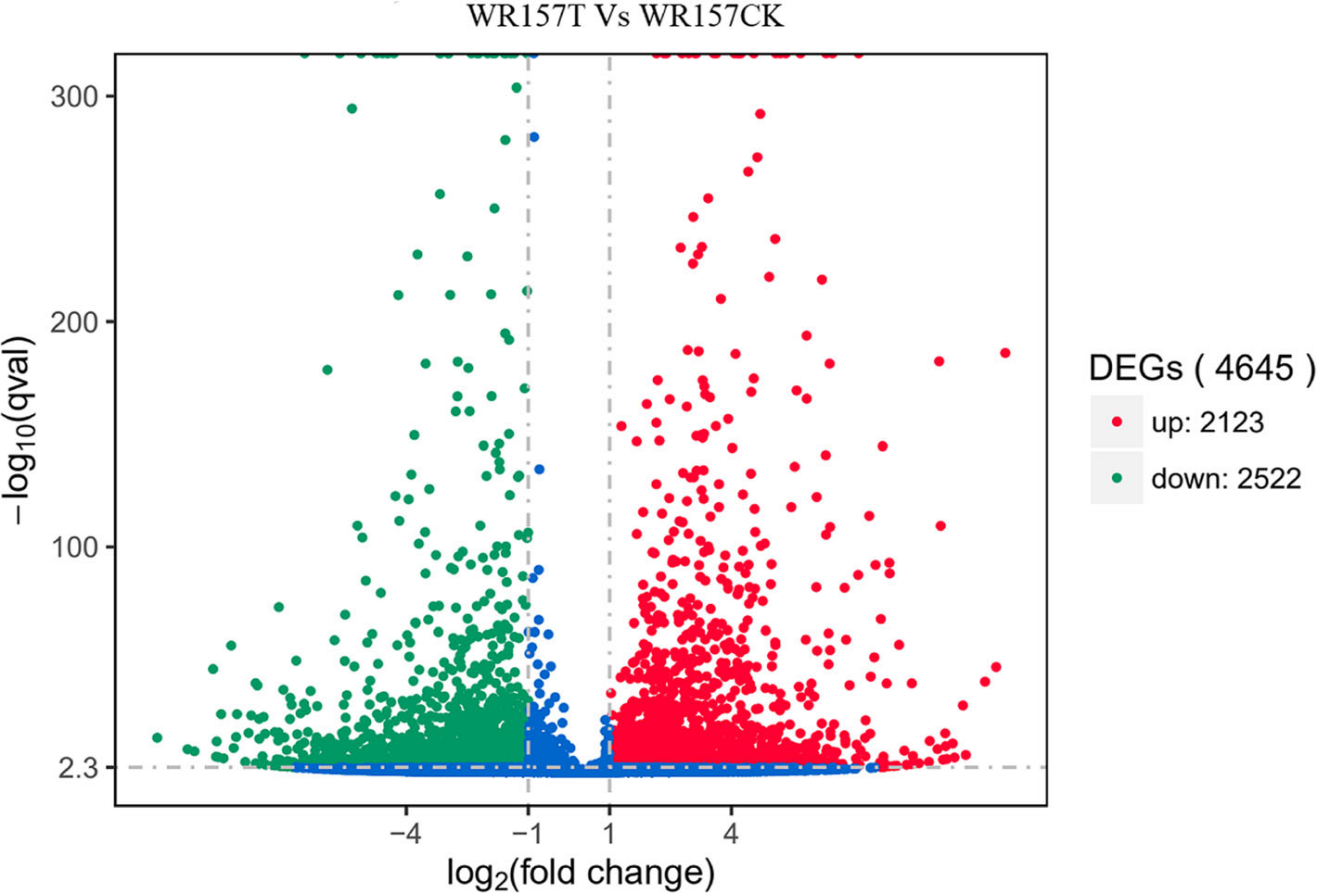
Upregulated (red) and downregulated (green) genes between WR157T and WR157CK. A total of 4645 differentially expressed genes (DEGs) were found between WR157T and WR157CK; 2123 DEGs were upregulated, and 2522 DEGs were downregulated

A total of 4645 putative DEGs were identified in treated groups (WR157T) compared to the control groups (WR157CK), with 2123 and 2522 DEGs upregulated and downregulated, respectively (Fig. 4, Additional file 6: Table S6). Among the 2123 upregulated DEGs, 1721 DEGs had been annotated, and 226 genes of 1721 DEGs had been named; ten genes out of the 226 DEGs were significantly upregulated (log2 fold change > 5.0). Among the 2522 downregulated DEGs, 1886 DEGs had been annotated, and 210 genes of 1886 DEGs had been named; 14 genes out of the 210 DEGs were significantly downregulated (log2 fold change <− 5) (Additional file 6: Table S6, Fig. 4).

### GO Term Enrichment Analysis Based on DEGs

**Fig. 5:**
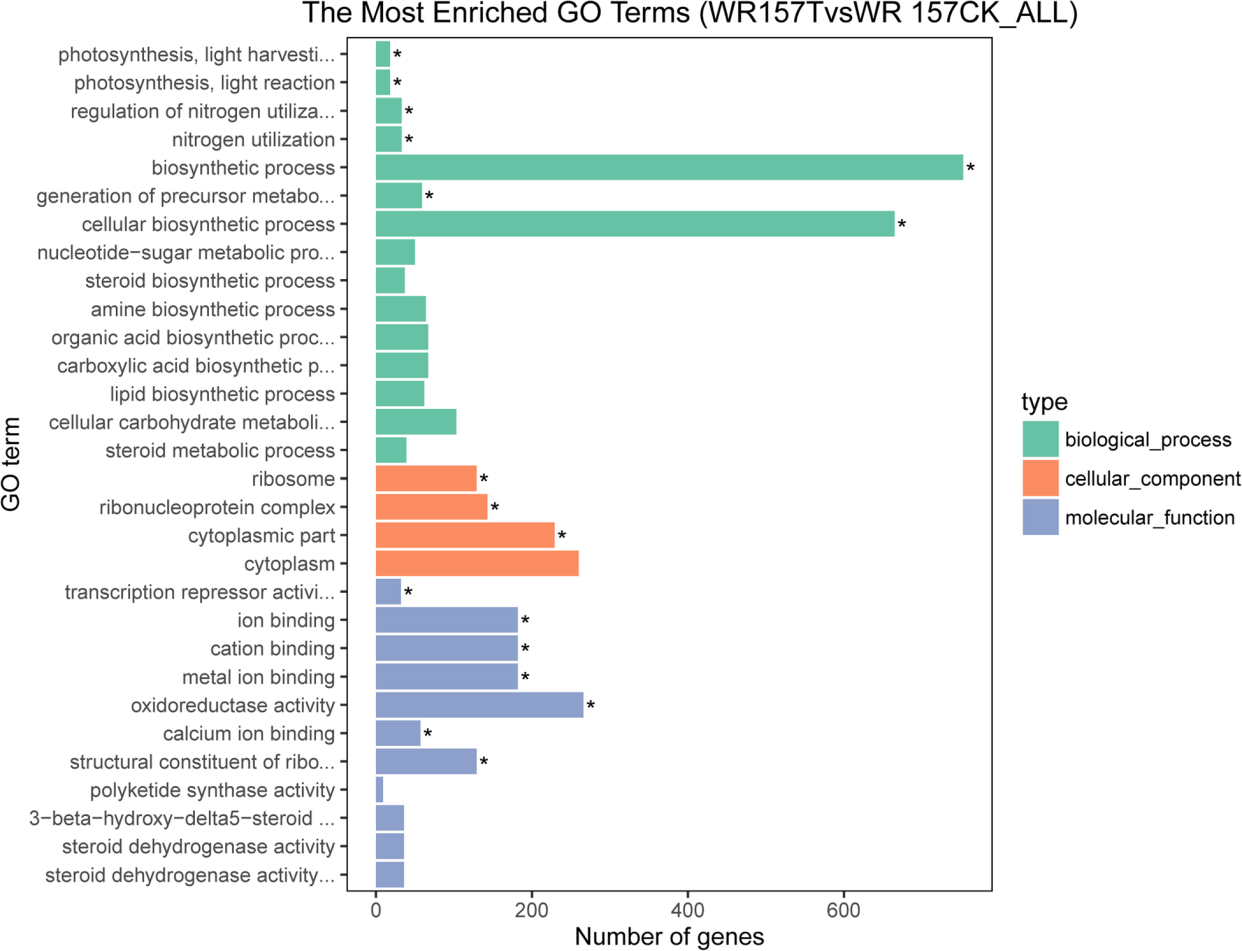
GO enriched term analysis of differentially expressed genes (DEGs). The asterisks represent significantly enriched GO terms. The histogram (green color) represents the DEGs enriched in biological processes, the orange color histogram represents the DEGs enriched in cellular components, and the blue color histogram represents the DEGs enriched in molecular functions

To identify the functions of the DEGs, a total of 1780 GO terms for all identified DEGs were enriched (Additional file 7: Table S7, Fig. 5). All upregulated genes were enriched on 1388 terms, and of these terms, 545 are for molecular function, 694 are for biological process, and 149 are for cellular component. However, 175 terms of 1388 terms for all upregulated genes were enriched significantly (Over_represented_pValue < 0.05). Of these, 113 terms were enriched in biological process, 51 terms were enriched in molecular function, and 11 terms were enriched in cellular component. All downregulated genes were enriched on 1566 terms, and of these terms, 612 terms are for molecular function, 772 terms are for biological process, and 182 terms are for cellular component (Fig. 5, Additional file 7: Table S7). However, 182 terms of 1566 terms enriched for all upregulated genes were enriched significantly (Over_represented_pValue < 0.05). Of these, 120 terms were enriched in biological process, 51 terms were enriched in molecular function, and 11 terms were enriched in cellular component.

**Fig. 6:**
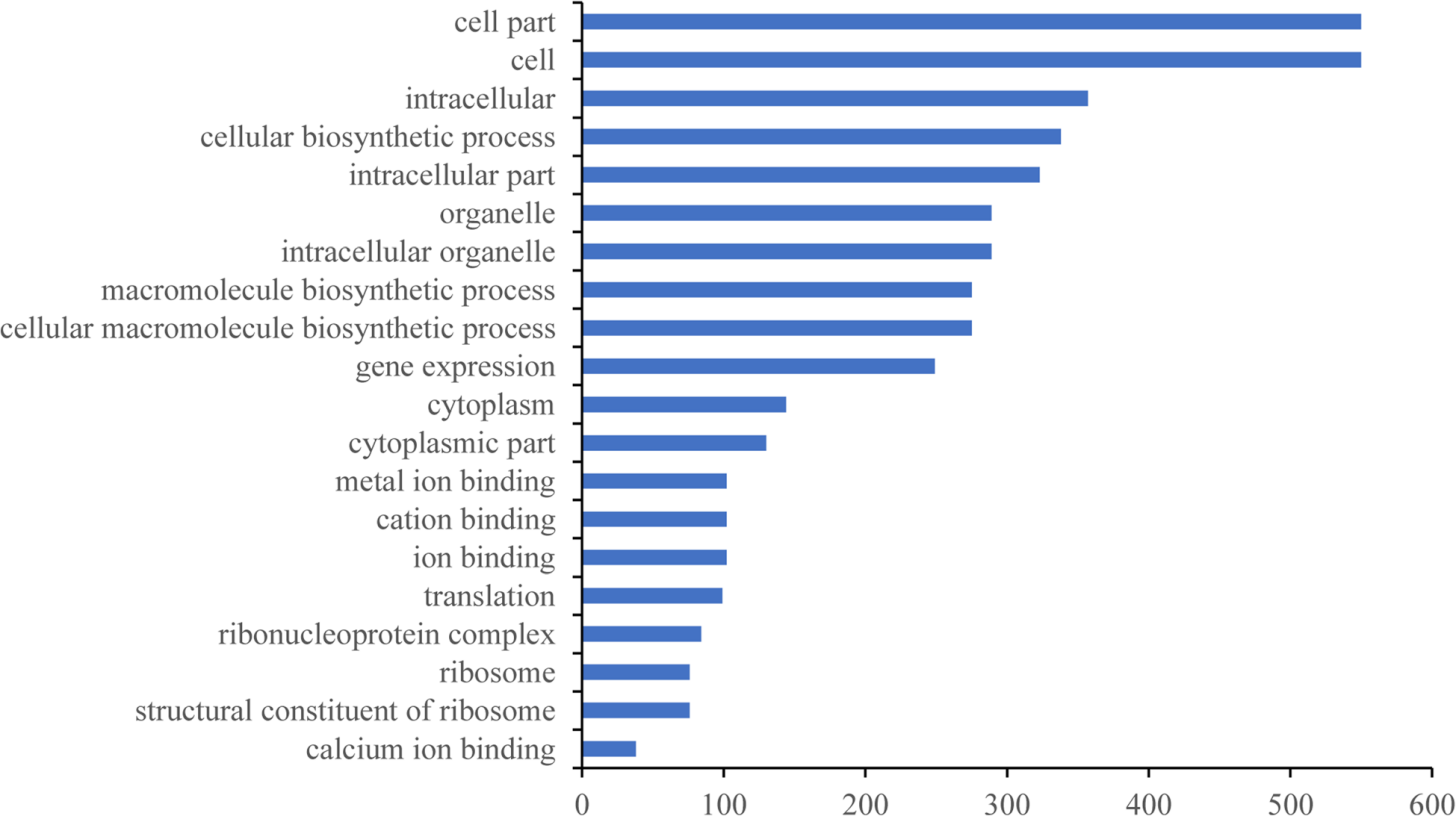
Number of significantly enriched DEGs associated with the 20 GO enriched terms. The ordinate value (Y-axis) represents the name of the previous 20 GO terms, and the X-axis value represents the number of DEGs enriched in the corresponding terms

Based on the 1780 terms enriched for all DEGs, 144 terms were enriched significantly (Over_represented_pValue < 0.05). The top 20 terms significantly enriched (*P* < 0.05) is mainly distributed in the cellular components and biological processes categories, which included cell parts, intracellular and cellular biosynthetic processes, intracellular parts, intracellular organelles, organelles, cellular macromolecule biosynthetic processes, macromolecule biosynthetic processes, gene expression, cytoplasm, and the ribosome (Fig. 6). GO:0019740 and GO:0006808 are involved in nitrogen utilization, which is in accordance with our findings relating the NRI to cold tolerance. GO:0009269 and GO:0009414 are related to the stress response, which suggested that these DEGs are related to cold tolerance. GO:0005623 and GO:0044464 showed enrichment of 1072 DEGs related to cellular components. GO:0016491 showed enrichment of 266 DEGs related to oxidoreductase activity, and GO:0016614, GO:0016616, GO:0016667, GO:0016627, and GO:0016715 were all related to oxidoreductase activity, which supports our use of the MDA and SOD evaluation indices (Additional file 8: Table S8).

### KEGG Pathway Analysis

**Fig. 7:**
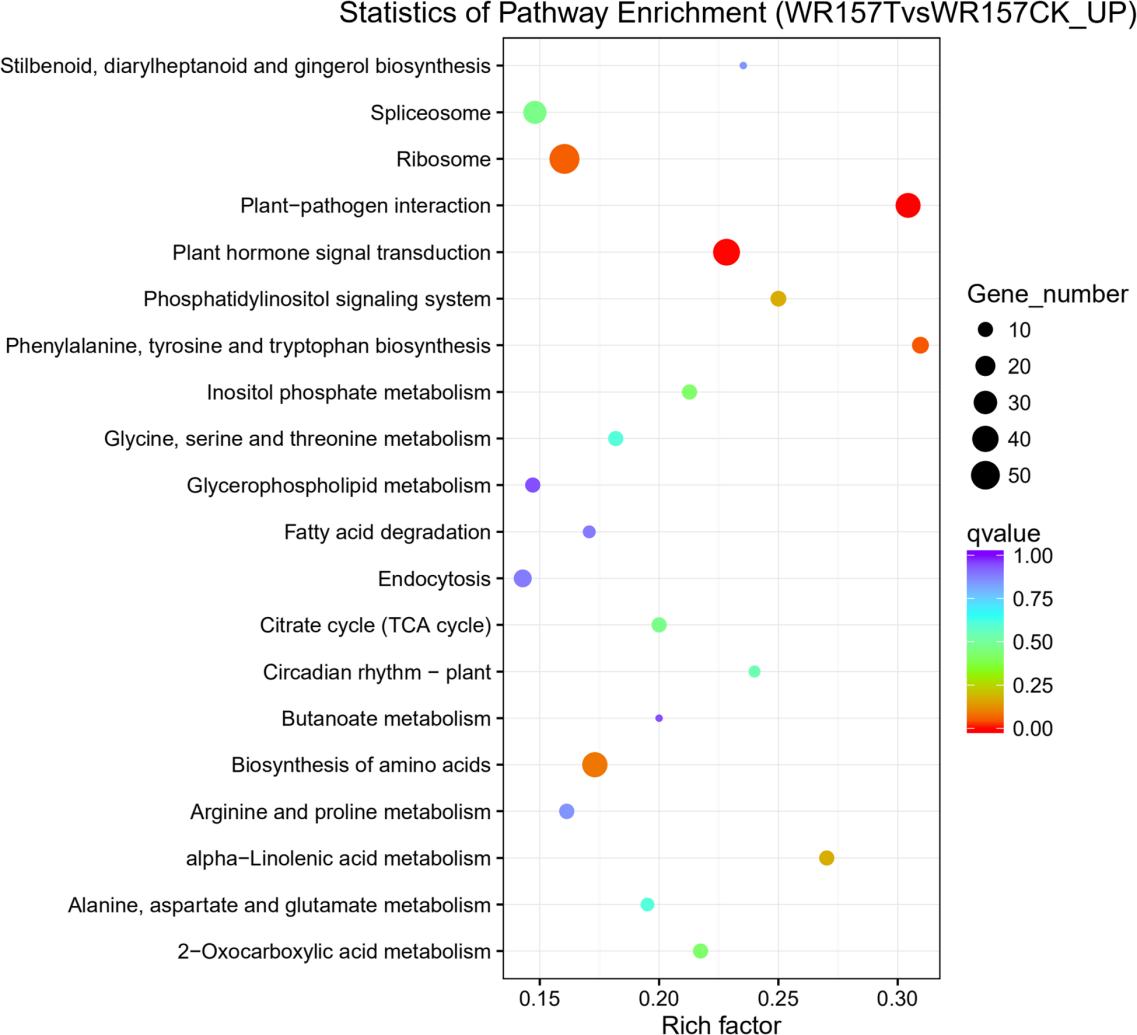
Pathway enrichment for upregulated DEGs in WR157T and WR157CK according to the KEGG analysis. The color of the circle represents the q value, and the size of the circle represents the number of genes enriched in the pathway. The rich factor represents the ratio between the number of enriched DEGs in a pathway and the number of annotated genes, higher values of the Rich factor mean a higher degree of enrichment

**Fig. 8:**
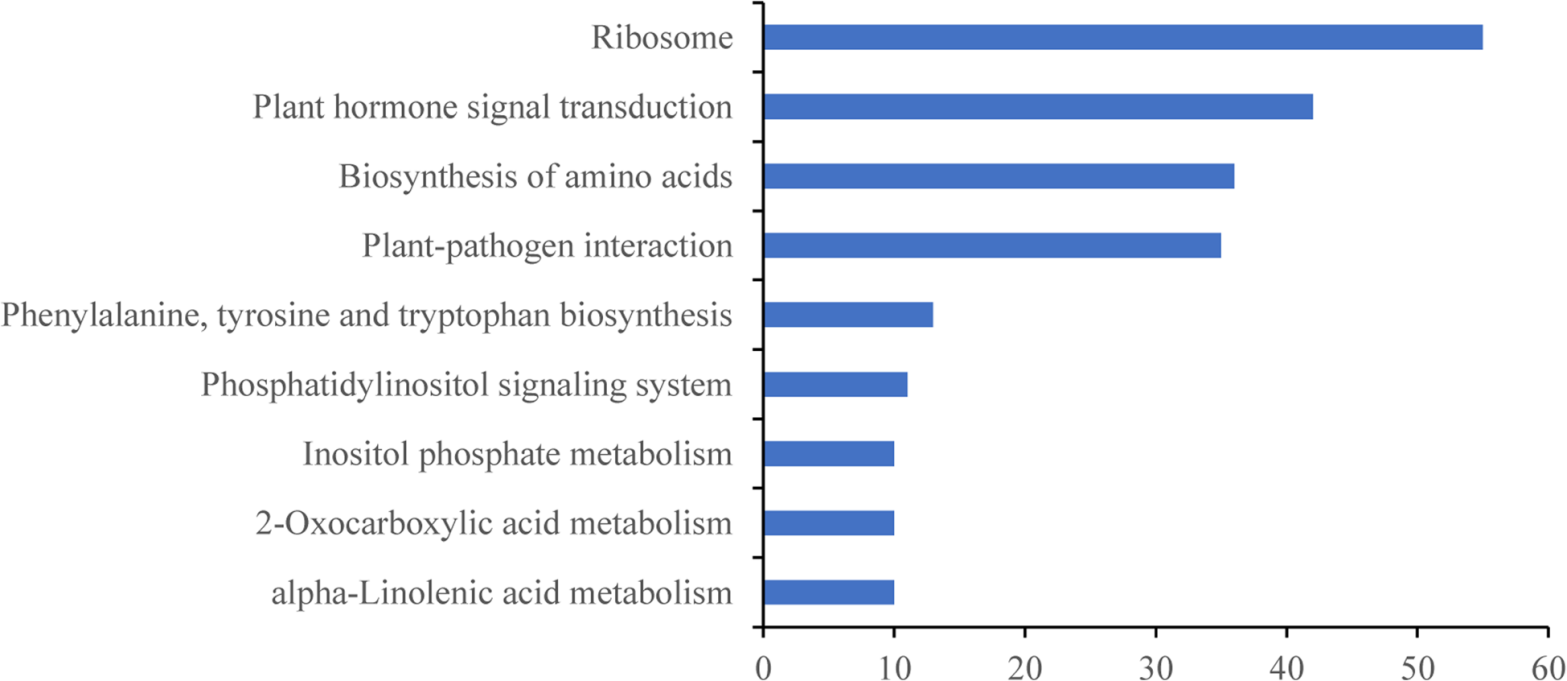
Number of significantly upregulated DEGs for each category in the KEGG pathways. The number indicates the enriched upregulated DEGs in the nine KEGG pathways

The Rich factor represents the ratio between the number of enriched DEGs in a pathway and the number of annotated genes, a higher Rich factors represent a higher degree of enrichment. KEGG (Kyoto Encyclopedia of Genes and Genomes) pathway analysis indicated that 911 of the 2123 upregulated DEGs fell into 98 KEGG categories and that 1103 of the 2522 downregulated DEGs were in 115 categories. Furthermore, among the upregulated KEGG pathway, the overrepresented KEGG pathway could be classified into 9 categories (Figs. 7, 8, and Additional file 9: Table S9). Among these categories, the ribosome pathway was overrepresented, with 55 DEGs (Fig. 7), suggesting that this pathway may regulate the expression of stress-induced genes. Plant hormone signal transduction pathways were overrepresented with 42 DEGs, so these pathways may also be related to the stress response. The pathway of the biosynthesis of amino acids were enriched with 36 DEGs; the plant-pathogen interaction pathway clustered with 35 DEGs; and the biosynthesis pathway of phenylalanine, tyrosine, and tryptophan (three essential aromatic amino acids) were enriched with 13 DEGs. The phosphatidylinositol signaling system pathway represented 11 DEGs, the inositol phosphate metabolism pathway was enriched with 10 DEGs, and the 2-oxocarboxylic acid metabolism pathway clustered with 10 DEGs.

## Discussion

### Evaluation of the Cold Tolerance of Weedy Rice

For each variety of weedy rice, the recovery index reflected its cold tolerance. In this study, we selected the comprehensive recovery index *D* based on the values of the SR, CRI, NRI, and LWD and selected RLWD7 to evaluate the cold-tolerance capacity. This approach proved more reliable than other traditional evaluation metrics. By using regression analysis, we showed that the NRI correlated with cold tolerance and thus may not only be integral to the mechanism of cold resistance but also serve as a marker of cold tolerance in rice. The GO term enrichment analysis of DEGs (Additional file 7: Table S7) revealed that GO:0019740 and GO:0006808 were related to nitrogen utilization and regulation, verifying that the NRI indeed plays a significant role in confronting cold damage. Moreover, the terms GO:0009765, GO:0019684, and GO:0015979, which are involved in photosynthesis, suggested that chlorophyll can also be an important evaluation index. The change in nitrogen content in leaves has been considered an evaluation standard against adverse natural conditions (Cao et al. 2018), and chlorophyll content can also be used as an indicator of abiotic stress (Lakra et al. 2015).

SOD was used as a physiological indicator of cold tolerance in weedy rice. We measured the SOD activity in 6 varieties and found a significant positive correlation between *pD* and SOD activity (Fig. 2) as well as between *pD* and MDA content (Fig. 3). These results are consistent with those of previous studies by Fu et al. (2007, 2009). Therefore, the *pD* value can be used to classify the cold tolerance of different varieties of rice, and SOD and MDA may also have critical influence on rice cold tolerance evaluation. GO:0016491, GO:0016614, GO:0016616, and GO:0016667 were related to oxidoreductase activity and may explain the efficacy of SOD activity and MDA contents as evaluation indices. Other physiological indicators may also indicate cold tolerance: GO:0009415 and GO:0009414 were related to water use (Additional file 8: Table S8), suggesting that cold-tolerance capacity has some connection with drought resistance.

### Transcriptome Analysis of the Cold-Tolerant Variety

All significantly up- or downregulated DEGs had some connection with stress resistance or oxidation resistance in plants (Xiao et al. 2018, Zhou et al. 2010). OS08G0189900 encodes the germin-like protein GmGLP10 and enhanced resistance capacity to bacteria (Zhang et al. 2017, Zhang et al. 2018). OS01G0566500 can relieve oxidative stress in plants and is related to the MDA and SOD produced under cold-stress conditions (Saito et al. 2010, Shen et al. 2018); OS08G0509100 is related to the synthesis of lipoxygenase and drives the antioxidant ability of soybean (*Glycine max)* (Soccio et al. 2018); OS01G0323600 allows the accumulation of S-adenosylmethionine synthetase transcripts under salt stress (Espartero et al. 1994); OS07G0592600 can regulate cold or drought resistance via hormones such as abscisic acid (ABA) (Du et al. 2012); and OS12G0147800 is a cold-induced gene in loquat (*Eriobotrya japonica)* (Song et al. 2017).

The KEGG pathway analysis sorted up- and downregulated DEGs into categories, of which the ribosome pathway was overrepresented (Fig. 8). This pathway may mainly regulate the expression of stress-induced genes. Ribosomes are important organelles made up of protein and rRNA (Ben-Shem et al. 2011). Subsequent rRNA modifications primarily take place in ribosomes, and these rRNA modifications react to environmental changes (cold, drought, and salt) in the development or disease process. This suggests that ribosome modifications may contribute to the translational control of gene expression under adverse conditions (Sloan et al. 2017). Plant hormone signal transduction pathways were also overrepresented, suggesting that the pathway is related to stress responses. The interactive nature of hormone signal transduction makes the mediation of defense responses possible. Verma et al. (2016) set forth the function of the major plant hormones and discussed the roles of some hormones, such as abscisic acid (ABA), salicylic acid, and ethylene, in response to abiotic stress. The biosynthesis of amino acids may also play a role in the response to stress. Cao et al. (2017) found that glycine increases cold tolerance in rice, and Miao et al. (2017) showed that specific amino acids are devoted to the cold adaptation of the *Micrococcus antarcticus* β-glucosidase BglU. The plant-pathogen interaction pathway may also play a role in cold resistance; Wu et al. (2014) showed that cold acclimation is related to disease resistance in Amur grape (*Vitis amurensis*). Phenylalanine, tyrosine, and tryptophan are essential components of proteins and precursors for various secondary metabolites that influence plant growth (Tzin and Galili 2010). Chen et al. (2014) found that aromatic amino acids play important roles in confronting mercury (Hg) stress in rice seedlings. The phosphatidylinositol signaling system and the inositol phosphate metabolism pathway are also known to be involved in stress resistance. Deng et al. (2018a, 2018b) found that phosphatidylinositol-hydrolyzing phospholipase is related to salt and drought stress in rice, and Kusuda et al. (2015) identified myo-inositol-3-phosphate synthase in response to salt tolerance in rice. The role of the 2-oxocarboxylic acid metabolism pathway deserves further study.

## Conclusion

Our comprehensive evaluation based on 5 morphological indices and 9 physiological indicators revealed outstanding levels of cold-tolerance capacity among weedy rice varieties from different regions and screened some genes related to cold tolerance via transcriptome analysis. Our results provide reliable evaluation methods for additional cold tolerance studies and revealed a few genes related to cold tolerance, which will help researchers breed cultivated rice varieties to increase their cold-tolerance capacity. This trait has the ability to increase seedling survival rate and growth, as well as future yields.

## Methods

### Planting of Materials

Thirty grains from each of 133 weedy rice varieties (Additional file 10: Table S10) were sown in an experimental basin in a greenhouse at the Chinese Academy of Sciences (Beijing, China) on 14 November 2017. The experimental basin was 66 × 35 × 22.5 cm, and it was filled with 5 kg of peat-based soil mixed with 10 L water. After the soil and water were mixed uniformly and thoroughly, the seeds were sown in the basin according to the standard row spacing of 2.5 cm and plant distance of 1 cm, and an air chute was in the middle 5 cm. Forty samples were sown in each basin, and each basin contained 2 rows of cold-tolerant control samples (Kongyu 131). Based on natural temperature change trends, two biological repetitions were included (sown on 14 and 21 November 2017, respectively). The control group of each variety was planted at the same time and was grown under the same conditions until the 3.5-leaf stage. The treatment group was then transferred to the natural low-temperature conditions, and the other conditions were consistent with those of the control group still in the original growing environment. The positive control Kongyu 131 is a strong cold-tolerant variety; it was planted, grown, treated and investigated under the same conditions as those of all the other varieties in our experiment.

### Cold-Tolerant Germplasm Evaluation

**Fig. 9:**
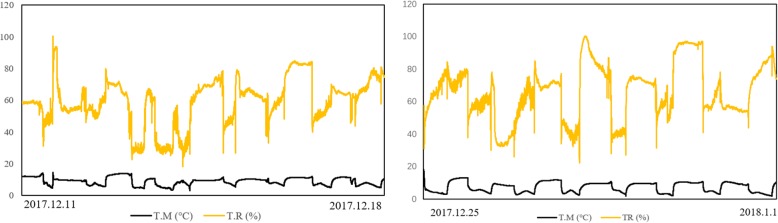
Temperature (black line) and humidity (yellow line) across the two biological replicates of the natural low-temperature treatment. The first replicate was treated from 11 December 2017 to 18 December2017, and the second biological replicate was treated from 25 December 2017 to 1 January 2018. Shown is the total across 8 d. The black line represents the change in natural low temperature. In the daytime, the natural temperature was higher than the temperature at night, but the highest temperature was only 14.5 °C, and the lowest temperature was only 1.8 °C

When the seedlings of the first biological replicate grew to the 3.5-leaf stage, the natural low-temperature treatment began and ran from 11 December 2017 to 18 December 2017. At 8:00 every day, the seedlings were transferred to a drought shed outside the greenhouse, and at 20:00, they were transferred back to the aisle in the greenhouse. The seedlings were thus exposed to the low-temperature treatment outside the greenhouse for 12 h daily, avoiding the night chilling temperature that would lead to the death of all seedlings. The temperature and humidity were monitored and recorded each minute using the instrument (MINI-TH, MTH1407120074A) throughout the experiment (Fig. 9, Table 4). The second replicate followed the same methods from 25 December 2017 to 1 January 2018. During this second period, the outdoor temperature was very low (Fig. 9, Table 4).

**Table 4:** The cold treatment and growth condtions for 133 rice varieties

Treament time	Treatment temperature				Treatment humidity				Light	Wind
(T.T)	(T.M °C)				(T.H)				(L)	(W.S)
12 h (D)/12 h (N)	(Max)	(Min)	(A)	(S)	(Max)	(Min)	(A)	(S)	Conformity	None
2017.12.11–2017.12.18	14.3	3.2	8.79	1707	65.6	23	213	9771.6	(Ck) Growth temperature	
2017. 12.25–2018.1.1	14.5	1.8	7.34	1430	68.8	20	178	10,758.3	23–25 °C	

Cold tolerance was evaluated according to standard methods for rice (Han et al. 2006, Han et al. 2018), with data collected at 0, 3, and 7 d after treatment, according to the leaf withering degree (LWD, Table 5) at each stage. The treated seedlings were then allowed to recover for 7 d, and the control and treatment groups were checked for seedling survival rate (CKs and Ts, respectively), chlorophyll content (CKc and Tc, respectively), and nitrogen content (CKn and Tn, respectively), which were measured using the plant nutrition tester (TYS-4 N, Top YunNong, Zhejiang). The instrument was set to zero, then leaves at the 3.5 leaves stage were chosen for the treatment and the control of each variety. Then, three test points at 3–5 cm away the leaf tip were used to check the chlorophyll content (CKc and Tc, respectively) and nitrogen content (CKn and Tn, respectively) three times; the results were attained based on the average of three test replicates. From these values, three factors were calculated: seedling survival rate (SR) = Ts / CKs, the chlorophyll content recovery index (CRI) = Tc / CKc, and the nitrogen content recovery index (NRI) = Tn / CKn. RLWD7 was measured after 7 d of recovery growth. The subordinate function μ was calculated using Eq. (1).

1 $$ \mu \left({x}_i\right)=\frac{x_i-{x}_{\mathrm{min}}}{x_{\mathrm{max}}-{x}_{\mathrm{min}}}i=1,2,\mathrm{3..........}n $$

**Table 5:** The investigation of Leaf Wither Degree under the cold stress conditions

Degree	LWD (Leaf Wither Degree)	Cold-tolerance Capacity
1	Normal leaf	Very strong
3	1/4 of total leaf area to wither	Strong
5	1/4–1/2 of total leaf area to wither	Middle strong
7	Greater than 2/3 of total leaf area to wither	Weak
9	Total plants wither	Very weak

In Eq. (1), *x*_*i*_ represents the ith comprehensive index, *x*_min_ represents the minimum for the ith comprehensive index, *x*_max_ represents the maximum for the ith comprehensive index, and the subordinate function values reflect the cold-tolerance capacity.

According to Eq. (2), the weight function *w*_*i*_ was calculated and represents the relative importance for the ith comprehensive index, and *p*_*i*_ represents the contribution for the ith comprehensive index. The comprehensive weights for the two comprehensive indices were 0.630 and 0.369, respectively.

2 $$ w{}_i={p}_i/\sum \limits_{i=1}^np,i=1,2,\mathrm{3..........}n $$

According to Eq. (3), the comprehensive evaluation value or recovery index *D* (Additional file 4: Table S4) for cold tolerance at the seedling stage was calculated.

3 $$ D=\sum \limits_{i-1}^n\left[\mu \left({x}_i\right)\ast {w}_i\right]i=1,2,\mathrm{3.........}n $$

The comprehensive evaluation parameter of cold tolerance resilience (*D*) was used to identify the cold-tolerance capacity of the different varieties.

### Cold Tolerance Physiological Index Assay

After the preliminary cold tolerance evaluation, six different cold-tolerant materials were selected: WR16, WR21, WR20, WR155, WR164, and WR157. After 3 d of cold treatment, leaf tissues were taken from control and treated seedlings. ELISA Kits and a BCA Protein Assay Kit for Protein Determination were used to check the enzyme abundance (Thermo-Fisher Scientific, Waltham, MA, USA) for nine physiological indices; each index used two ELISA Kits: SOD activity, CAT activity, TPI activity, MDA content, Pro content, ATPase activity, ABA content, Chl content, and GSH content. The protocol for the ELISA to determine the physiological indices was as follows: leaf tissue of each variety was removed at the 3-d stage after cold treatment, samples in PBS were amplified and mixed according to the proportion of 10 mg/100 μL, and 1 ml was equal to 0.1 g samples. Then the original OD value of each replicates of each sample were read using the a Rayto RT-6100 instrument, and the standard curve of each indices were made according to BCA Kit, then the Converted concentration of each were calculated, and three repetitions of each variety were included.

### Transcriptome Sequence Assay

The cold-tolerant sample WR157 was selected based on the ELISA assay results for further analysis. At 3 d of treatment, leaf tissues were collected, and RNA was extracted and checked using an Agilent 2100 Bioanalyzer (Agilent, Santa Clara, CA, USA) for the control and treated samples. The samples were considered to be of adequate concentration and quality when the value of OD260/280 was greater than 1.87 and less than 2.09, the value of OD260/230 was greater than 2.02 and less than 2.51, the value of 28S/18S was greater than 1.1 and less than 1.6, and the value of RIN was greater than 7.5 and less than 8.2. The qualified RNA was constructed into a library and sequenced using a high-throughput sequencing platform HiSeq™ 2500 (Illumina, San Diego, CA, USA). The sequencing data QC was then checked by the Phred quality score and Q value, with sequence comparison and transcript splicing carried out by TopHat2 and Cufflinks software (Kim et al. 2013; Trapnell et al. 2010). Gene quantification analysis was conducted by introducing FPKM (fragments per kilobase of exon model per million mapped reads). Gene Ontology (GO), http://www.geneontology.org/) and KEGG (Kyoto Encyclopedia of Genes and Genomes; http://www.kegg.jp) analyses were also conducted (Young et al. 2010; Kanehisa et al. 2008). KEGG (Kanehisa et al. 2008) is a database resource for understanding high-level functions and utilities of the biological system, such as the cell, the organism and the ecosystem, from molecular-level information, especially large-scale molecular datasets generated by genome sequencing and other high-throughput experimental technologies (http://www.genome.jp/kegg/). We used KOBAS (Mao et al., 2005) software to test the statistical enrichment of differential expression genes in KEGG pathways. KEGG (Kyoto Encyclopedia of Genes and Genomes; http://www.kegg.jp) analyses were also conducted (Young et al. 2010; Kanehisa et al. 2008) and identified the major terms of biochemical metabolic pathways and signal transduction pathways participated for the DEGs based on the corrected *P*-value, when corrected *P*-value < 0.05, the terms were regarded as significantly enrich. The genome of the *Japonica* rice cultivar Nipponbare was used as the reference genome.

### Statistical Analysis

PCA (principal component analysis), correlations and significance analysis were performed using SPSS 17.0. The recovery index refers to the proportion between the index value under low-temperature stress and the normal temperature when the treated seedlings had recovered for 7 d, and a significant correlation means *P* < 0.05.

## Supplementary information

**Additional file 1: Table S1.** Five single index value for 133 weedy rice and the control variety Kongyu131.

**Additional file 2: Table S2.** The value of comprehensive index for each variety.

**Additional file 3: Table S3.** The subordinate function value of every variety.

**Additional file 4: Table S4.** the comprehensive evaluation value (*D*) of each variety.

**Additional file 5: Table S5.** The value of the nine physiological index.

**Additional file 6 **: **Table S6.** The differential expression genes between WR157T and WR157CK.

**Additional file 7: Table S7.** GO enriched term analysis of differential expression genes (DEGs).

**Additional file 8: Table S8.** GO enriched terms related to the selected evaluation index.

**Additional file 9: Table S9.** Pathway enrichment for upregulated-DEGs in WR157T and WR157CK according to KEGG analysis.

**Additional file 10: Table S10.** The name for 133 weedy rice samples and the control.

## Data Availability

All data generated or analyzed during this study are included in this published article and its Additional files. The sequencing dataset used in the study is available in the NCBI repository (SUB6401025) for WR157CK and WR157T.

## Abbreviations

ABA: Abscisic acid
ATPase: ATP synthase
CAT: Catalase (CAT)
Chl: Chlorophyll
CR: Contribution rate
CRI: Chlorophyll recovery index
DEGs: Differential expression genes
GO: Gene ontology
GSH: Glutathione
KEGGs: Kyoto Encyclopedia of Genes and Genomes
LWD7: Leaf withering degree after 7 d of cold treatment
MDA: Malondialdehyde
NRI: Nitrogen recovery index
PCA: Principal component analysis
Pro: Proline
RLWD7: LWD after 7 d of recovery growth
SOD: Superoxide dismutase (SOD)
SR: Seedling survival rate
TPI: Triosephosphate isomerase

## References

[CR1] Agarwal PK, Agarwal P, Reddy MK, Sopory SK (2006). Role of DREB transcription factors in abiotic and biotic stress tolerance in plants. Plant Cell Rep.

[CR2] Bartels D, Sunkar R (2005). Drought and salt tolerance in plants. CRC Crit Rev Plant Sci.

[CR3] Ben-Shem A, Garreau de Loubresse N, Melnikov S (2011). The structure of the eukaryotic ribosome at 3.0 Å resolution. Science.

[CR4] Xiaochuang Cao, Chu Zhong, Chunquan Zhu, Junhua Zhang, Lianfeng Zhu, Lianghuan Wu, Qianyu Jin (2019). Variability of leaf photosynthetic characteristics in rice and its relationship with resistance to water stress under different nitrogen nutrition regimes. Physiologia Plantarum.

[CR5] Cao XC, Zhong C, Zhu LF (2017). Glycine increases cold tolerance in rice via the regulation of N uptake, physiological characteristics, and photosynthesis. Plant Physiol Biochem.

[CR6] Chen HZ, Jiang WP, Xie W, Liu XP (2015). Advances and suggestion of cold tolerance of Rice. Hubei Agric Sci.

[CR7] Chen YA, Chi WC, Trinh NN (2014). Transcriptome profiling and physiological studies reveal a major role for aromatic amino acids in mercury stress tolerance in rice seedlings. PLoS One.

[CR8] Choi HI, Hong JH, Ha JO, Kang JY, Kim SY (2000). ABFs, a family of ABA responsive element binding factors. J Biol Chem.

[CR9] Cruz RP, Sperotto RA, Cargnelutti D (2013). Avoiding damage and achieving cold tolerance in rice plants. Food Energy Secur.

[CR10] Dametto A, Sperotto RA, Adamski JM (2015). Cold tolerance in rice germinating seeds revealed by deep RNA-seq analysis of contrasting Indica genotypes. Plant Sci.

[CR11] Deng C, Ye H, Fan M (2017). The rice transcription factors *OsICE* confer enhanced cold tolerance in transgenic Arabidopsis. Plant Signal Behav.

[CR12] Deng QW, Luo XD, Chen YL (2018). Transcriptome analysis of phosphorus stress responsiveness in the seedlings of Dongxiang wild rice. Biol Res.

[CR13] Deng Xianjun, Yuan Shu, Cao Huasheng, Lam Sin Man, Shui Guanghou, Hong Yueyun, Wang Xuemin (2018). Phosphatidylinositol-hydrolyzing phospholipase C4 modulates rice response to salt and drought. Plant, Cell & Environment.

[CR14] do Amaral MN, Arge LW, Benitez LC (2016). Comparative transcriptomics of rice plants under cold, iron, and salt stresses. Funct Genomics.

[CR15] Du H, Wu N, Fu J (2012). A GH3 family member, *OsGH3-2,* modulates auxin and abscisic acid (ABA) levels and differentially affects drought and cold tolerance in rice. J Exp Bot.

[CR16] Espartero J, Pintor-Toro JA, Pardo JM (1994). Differential accumulation of S-adenosylmethionine synthetase transcripts in response to salt stress. Plant Mol Biol.

[CR17] Farooq M, Wahid A, Kobayashi ND, Fujita SMA (2009). Review article plant drought stress: effects, mechanisms and management. Agron Sustain Dev.

[CR18] Fu TL, Ma J, Li M (2009). Comprehensive evaluation and screening identification indexes of cold tolerance at seedling stage in Rice. Southwest China J Agric Sci.

[CR19] Fu TL, Ma J, Wang HZ (2007). Comprehensive evaluation and screening identification indexes of cold tolerance at flowering stage in rice. Southwest China J Agric Sci.

[CR20] Guan S, Xu Q, Ma D (2018). Transcriptomics profiling in response to cold stress in cultivated rice and weedy rice. Gene.

[CR21] Han B, Wang J, Li YF (2018) Identification of quantitative trait loci associated with drought tolerance traits in Rice (*Oryza sativa L*.) under PEG and Field drought stress. Euphytica 214:74

[CR22] Han LZ, Wei XH (2006). Descriptive specification and data standard of rice germplasm resources.

[CR23] Kanehisa M, Araki M, Goto S (2008). KEGG for linking genomes to life and the environment. Nucleic Acids Res.

[CR24] Kim D, Pertea G, Trapnell C (2013). TopHat2: accurate alignment of transcriptomes in the presence of insertions, deletions and gene fusions. Genome Biol.

[CR25] Kuroki M, Saito K, Matsuba S (2007). A quantitative trait locus for cold tolerance at the booting stage on rice chromosome 8. Theor Appl Genet.

[CR26] Kusuda H, Koga W, Kusano M (2015). Ectopic expression of myo-inositol 3-phosphate synthase induces a wide range of metabolic changes and confers salt tolerance in rice. Plant Sci.

[CR27] Lakra N, Nutan KK, Das P (2015). A nuclear-localized histone-gene binding protein from rice (*OsHBP1b*) functions in salinity and drought stress tolerance by maintaining chlorophyll content and improving the antioxidant machinery. J Plant Physiol.

[CR28] Li J, Pan Y, Guo H (2018). Fine mapping of QTL qCTB10-2 that confers cold tolerance at the booting stage in rice. Theor Appl Genet.

[CR29] Li YB, Kang M, Song W (2015). Effects of low temperature on physiological indexes of cold tolerance in progeny of cultivated rice transferred the total genomic DNA of chilling wild rice. Crop Res.

[CR30] Ma FF, Su YB, Zhang B (2016). Cloning and prokaryotic expression of TPI gene maize. J Shanxi Agric Univ.

[CR31] Ma Y, Dai X, Xu Y (2015). *COLD1* confers chilling tolerance in rice. Cell.

[CR32] Mao X, Cai T, Olyarchuk JG, Wei L (2005). Automated genome annotation and pathway identification using the KEGG Orthology (KO) as a controlled vocabulary. Bioinformatics.

[CR33] Miao LL, Fan HX, Qu J (2017). Specific amino acids responsible for the cold adaptedness of micrococcus antarcticus β-glucosidase BglU. Appl Microbiol Biotechnol.

[CR34] Moraes de Freitas GP, Basu S, Ramegowda V, Thomas J, Benitez LC, Braga EB, Pereira A (2019). Physiological and transcriptional responses to low-temperature stress in rice genotypes at the reproductive stage. Plant Signal Behav.

[CR35] Peng Y, Arora R, Li G, Wang X, Fessehaie A (2008). Rhododendron catawbiense plasma membrane intrinsic proteins are aquaporins, and their over-expression compromises constitutive freezing tolerance and cold acclimation ability of transgenic Arabidopsis plants. Plant Cell Environ.

[CR36] Saito K, Hayano-Saito Y, Kuroki M (2010). Map-based cloning of the rice cold tolerance gene *Ctb1*. Plant Sci.

[CR37] Shen Y, Li J, Shi S (2018). Application of carotenoid to alleviate the oxidative stress caused by phenanthrene in wheat. Environ Sci Pollut Res.

[CR38] Sloan KE, Warda AS, Sharma S (2017). Tuning the ribosome: the influence of rRNA modification on eukaryotic ribosome biogenesis and function. RNA Biol.

[CR39] Soccio Mario, Laus Maura, Flagella Zina, Pastore Donato (2018). Assessment of Antioxidant Capacity and Putative Healthy Effects of Natural Plant Products Using Soybean Lipoxygenase-Based Methods. An Overview. Molecules.

[CR40] Somerville C (1995). Direct tests of the role of membrane lipid composition in low-temperature-induced photoinhibition and chilling sensitivity in plants and cyanobacteria. Proc Natl Acad Sci U S A.

[CR41] Song H, Wang X, Hu W (2017). A cold-induced phytosulfokine peptide is related to the improvement of loquat fruit chilling tolerance. Food Chem.

[CR42] Thomashow MF (1999). Plant cold acclimation: freezing tolerance genes and regulatory mechanisms. Annu Rev Plant Physiol Plant MolBiol.

[CR43] Trapnell C, Williams BA, Pertea G (2010). Transcript assembly and quantification by RNA-Seq reveals unannotated transcripts and isoforms witching during cell differentiation. Nat Biotechnol.

[CR44] Tzin V, Galili G (2010). New insights into the shikimate and aromatic amino acids biosynthesis pathways in plants. Mol Plant.

[CR45] Uno Y, Furihata T, Abe H, Yoshida R, Shinozaki K (2000). Arabidopsis basic leucine zipper transcription factors involved in an ABA-dependent signal transduction pathway under drought and high-salinity conditions. Proc Natl Acad Sci.

[CR46] Verma V, Ravindran P, Kumar PP (2016). Plant hormone-mediated regulation of stress responses. BMC Plant Biol.

[CR47] Wu J, Zhang Y, Yin L (2014). Linkage of cold acclimation and disease resistance through plant-pathogen interaction pathway in Vitis amurensis grapevine. Funct Integr Genomics.

[CR48] Xiao N, Gao Y, Qian H (2018). Identification of genes related to cold tolerance and a functional allele that confers cold tolerance. Plant Physiol.

[CR49] Xiao YL, Wang ZQ, Lei JG (2014). A new method for accurate identification and evaluation of cold tolerance of rice at bud stage. Acta Agric Jiangxi.

[CR50] Yang ZQ, Yang CG, Tang CF (2010). Evaluation of cold tolerance at the booting stage and correlation analysis among cold tolerant traits for japonica landrace rice. J Plant Genet Resour.

[CR51] Young MD, Wakefield MJ, Smyth GK (2010). Gene ontology analysis for RNA-seq: accounting for selection bias. Genome Biol.

[CR52] Zhang T, Zhao X, Wang W (2012). Comparative transcriptome profiling of chilling stress responsiveness in two contrasting rice genotypes. PLoS One.

[CR53] Zhang Y, Wang X, Chang X (2018). Overexpression of germin-like protein GmGLP10 enhances resistance to Sclerotinia sclerotiorum in transgenic tobacco. Biochem Biophys Res Commun.

[CR54] Zhang Z, Li J, Pan Y (2017). Natural variation in CTB4a enhances rice adaptation to cold habitats. Nat Commun.

[CR55] Zhou L, Zeng Y, Zheng W (2010). Fine mapping a QTL qCTB7 for cold tolerance at the booting stage on rice chromosome 7 using a near-isogenic line. Theor Appl Genet.

[CR56] Zhou Y, Yang P, Cui F (2016). Transcriptome analysis of salt stress responsiveness in the seedlings of Dongxiang wild Rice. PLoS One.

